# The Case for Mass Treatment of Intestinal Helminths in Endemic Areas

**DOI:** 10.1371/journal.pntd.0004214

**Published:** 2015-10-22

**Authors:** Joan Hamory Hicks, Michael Kremer, Edward Miguel

**Affiliations:** 1 Center for Effective Global Action, University of California, Berkeley, Berkeley, California, United States of America; 2 Department of Economics, Harvard University, Cambridge, Massachusetts, United States of America; University of Queensland, AUSTRALIA

## Abstract

Two articles published earlier this year in the *International Journal of Epidemiology* [1,2] have re-ignited the debate over the World Health Organization’s long-held recommendation of mass-treatment of intestinal helminths in endemic areas. In this note, we discuss the content and relevance of these articles to the policy debate, and review the broader research literature on the educational and economic impacts of deworming. We conclude that existing evidence still indicates that mass deworming is a cost-effective health investment for governments in low-income countries where worm infections are widespread.

## Re-analysis of a School-Based Deworming Program in Kenya

A key question in this policy debate is whether deworming increases school attendance (and improves other life outcomes), and if so, whether distributing deworming pills through schools is the most cost-effective way of doing so. An earlier study evaluating a school-based deworming program in rural Kenya [3] had three main findings:

1. Deworming reduces worm infections in both treated children, and untreated children living nearby (through reduced disease transmission).
2. Deworming improves school attendance for treated and nearby untreated children.
3. Deworming does not improve academic test scores for children in this age group.

We have made the data, data manual, and replication manual with fully updated tables from this article publicly available to numerous other scholars since 2007, many of whom apparently used the data for replication exercises in their graduate classes. None of these scholars, or the students carrying out replications in their classes, contacted us to discuss the differences between the original and updated results, nor did any apparently believe that they merited publication.

The data was recently re-analyzed by an independent team of investigators. In a “pure replication”, the re-analysis authors use the methods of the original paper [1]. Although various errors and typos are corrected, all three main findings are affirmed. In particular, we argue in a Commentary in the *International Journal of Epidemiology* [4] that the “pure replication” confirms substantial reductions in worm infections and increases in school attendance due to deworming, for both treated and nearby untreated individuals.

Table 1, reproduced from [4], displays the results of the original study [3] and of this re-analysis [1] side by side for worm infections and school participation. This table makes it immediately clear how similar the original and updated results are. The only difference in key findings between the original paper and the re-analysis is that impacts on untreated individuals do not reach distances quite as far as originally believed: worm infections are reduced among schoolchildren living within 3 km of treated individuals, but not much beyond that, although the original paper estimated benefits out to a distance of 6 km due to a coding error. The overall externality effect in the 3–6 km range is less precisely estimated in the re-analysis.

**Table 1:** Deworming treatment effect estimates

	Treatment minus control (1)		Within-school externality (2)		Externality to 3 km (3)		Externality from 3 to 6 km (4)	
	Original	Updated	Original	Updated	Original	Updated	Original	Updated
Worm infections	-0.25**	-0.31**	-0.12*	-0.18**	-0.11**	-0.09**	-0.10**	-0.05
School participation	0.055**	0.055**	0.056**	0.056**	0.029**	0.023*	-0.01	-0.04

Notes: Estimates from the original article [3] and updated estimates from the re-analysis [1]. Moderate-heavy intestinal worm infection is the dependent variable in the first row, and the school participation rate is the dependent variable in the second row. The estimated effect is: the difference between treatment schools and control schools in Column 1; the within-school externality effect for untreated pupils in the treatment schools in Column 2; cross-school average externality effect for schools within 3 kilometers of treatment schools in Column 3, and between 3 to 6 kilometers of treatment schools in Column 4. Data previously reported in Ref. [4]. Stars reflect: “**” P-value < 0.05, “*” P-value < 0.10.

A second re-analysis applies alternative statistical methods to the same data [2]. This piece makes a series of non-standard analytical decisions and errors, many of which run counter to the authors’ own pre-analysis plan [5]. The following are four examples:

1. The original study covered two years and as the re-analysis authors’ own power calculations make clear, using both years of data is necessary for adequate statistical power [5]. The authors arbitrarily divide the data into two separate one-year experiments, dramatically reducing statistical power and making it unlikely that estimates will be statistical significant.
2. Rather than weight every individual child equally (or every school attendance check equally), the authors weight each school equally, such that a child in large population school has much less weight in the analysis than a child in a small population school. This is a non-standard statistical approach in both public health research and in economics with no natural interpretation or justification. We raise this issue in [4] but the replication authors do not offer a defense of this analytical approach in their response [6].
3. The deworming treatment measure is misdefined to include periods before treatment even began, and before it was even supposed to have begun (and thus the replication authors’ claim that this is an intention-to-treat analysis is incorrect). Defining people as treated before anyone received a pill or worm prevention lecture is an error, and simply adds unnecessary “noise” to the treatment variable.
4. The authors do not account for the epidemiological externalities (spillovers) from deworming treatment, although they confirm that these exist in their earlier work [1], and thus the treatment effects they estimate are lower bounds on the true effects.

These are all questionable analytical choices, and we discuss elsewhere [4] that it is only when some of these analytical errors are made in combination—in failing to pool the data, weighting observations, defining treatment, and ignoring treatment externalities—that deworming impact estimates on school attendance are not statistically significant.

The re-analysis authors raise concerns about the stepped-wedge research design of the original trial, arguing that there may be a link between school participation levels and the number of attendance observations collected that is related to treatment status, and use this as a justification for splitting the two-year experiment into multiple sub-experiments. We show elsewhere that there is no statistical basis for this claim [4], and the replication authors fail to offer a rebuttal in their response [6].

One solution to any concerns over the stepped-wedge design is to restrict analysis to the Group 1 schools (which were treatment schools in both years 1 and 2 of the study) and the Group 3 schools (controls in both years), while excluding the Group 2 schools that switched from control to treatment status between years 1 and 2. In a population-weighted cluster summary analysis presented in Table 2, this approach yields large, positive and statistically significant impacts of deworming on school participation (coefficient estimate +5.94 percentage points, P-value = 0.036), despite dropping one-third of the study sample.

**Table 2:** Deworming treatment effects on school participation, cluster summary analysis of Group 1 versus Group 3.

	Dependent variable: School participation rate	
	Difference	P-value
Treatment indicator and year defined as in [3], Miguel and Kremer (2004)	5.94**	[0.036]
Treatment indicator and year defined as in [2], Davey et al. (2015)	5.80**	[0.050]

This analysis is based on the top left panel of Table 2 in [2]. All analysis includes only individuals in Group 1 and Group 3 schools, and eligible, non-transferring pupils, for both 1998 and 1999. Regression includes a Year 2 indicator variable, weights each school by its total pupil population, and clusters the disturbance terms by school. P-values are in square brackets and stars reflect: “**” P-value < 0.05, “*” P-value < 0.10.

This result provides further evidence that deworming robustly increases school attendance in this Kenyan dataset.

## The Broader Evidence on the Impact of Deworming

Although recent popular press coverage has given the impression that the case for deworming rests on just one study [3], there are in fact multiple studies documenting the educational and economic impact of deworming. A recent paper [7] reviews the literature, which includes:

- A study showing that infant children who lived in these same communities where the randomized Kenyan deworming program was conducted, and thus were exposed to epidemiological spillovers (but not treated directly), show large cognitive test score improvements ten years later [8]. The magnitude of the effect is 0.2 to 0.3 standard deviation units, which is equivalent to between 0.5 to 0.8 years of schooling, and effects are nearly twice as large for children with an older sibling likely to have received medication directly. This provides additional empirical evidence of large positive deworming treatment externalities.
- A recent study of a separate randomized community-based deworming program in Uganda, showing that children exposed to more years of deworming have higher test scores in literacy and numeracy 7 to 8 years later, with effect sizes of 0.2 to 0.4 standard deviation units [9].
- A long-term follow-up of the Kenyan study, which finds that 10 years after deworming, Kenyan women who were dewormed for more years as girls were 25% more likely to have attended secondary school, and men who were dewormed for more years as boys worked 17% more hours and had better labor market outcomes, including higher earnings [10].
- A historical study of a deworming campaign in the southern United States in the early 1900’s, showing treatment lead to increased school enrollment and attendance for children, and improved literacy and boosted income by 17% for adults who were treated as children [11]. Given the nature of the historical intervention studied, this is a carefully executed difference-in-difference design, rather than an RCT.

The recently published Cochrane Review on deworming [12] does not include any of these papers. The historical paper on the U.S. South is excluded because it is not a randomized controlled trial, which is understandable under the rules of the Review, but since it uses a credible research design and has an extremely long follow-up period, it deserves weight in any assessment of whether deworming is an appropriate policy. The first three are excluded because they are as yet unpublished (although perhaps not for long). Taken together, these studies provide the best evidence on the long-run educational and labor market impacts of deworming, so their exclusion–even if justified under the rules of the Review–greatly limits the value of the recent Cochrane Review [12] for public policy choices.

The Cochrane Review [12] also opts to split the dataset in [3] into separate year-by-year comparisons, similar to the re-analysis in [2], rather than pooling data across both years in its meta-analysis. As we argue above, we believe this is an inappropriate analytical decision, and one that contributes to the Review’s conclusion that there is limited evidence linking deworming to school attendance.

In the recent paper mentioned above [7], we discuss the growing body of evidence on the impacts of deworming on educational and economic outcomes, and outline the circumstances under which mass deworming is an appropriate policy decision. Given the strength of this growing body of evidence, and the very high expense of the Cochrane-preferred “test and treat” option for worm control in areas where a large share of the population is infected, we conclude that mass deworming treatment should remain a policy priority in endemic regions.
